# Cerebellar hemorrhages in patients with Dutch-type hereditary cerebral amyloid angiopathy

**DOI:** 10.1177/17474930211043663

**Published:** 2021-09-10

**Authors:** S Voigt, PC de Kruijff, EA Koemans, I Rasing, ES van Etten, GM Terwindt, MJP van Osch, MA van Buchem, MAA van Walderveen, MJH Wermer

**Affiliations:** 1 Department of Neurology, Leiden University Medical Center, Leiden, the Netherlands; 2 Department of Radiology, Leiden University Medical Center, Leiden, the Netherlands

**Keywords:** Cerebral amyloid angiopathy, intracerebral hemorrhage, cerebellum, microbleeds, D-CAA

## Abstract

**Background:**

Recent studies suggest that superficially located cerebellar intracerebral hemorrhage (ICH) and microbleeds might point towards sporadic cerebral amyloid angiopathy (CAA).

**Aims:**

We investigated the proportion of cerebellar ICH and asymptomatic macro- and microbleeds in Dutch-type hereditary CAA (D-CAA), a severe and essentially pure form of CAA.

**Methods:**

Symptomatic patients with D-CAA (defined as ≥1 symptomatic ICH) and presymptomatic D-CAA mutation-carriers were included. We assessed magnetic resonance imaging scans for symptomatic (cerebellar) ICH and asymptomatic cerebellar macro- and microbleeds according to the STRIVE-criteria. Location was assessed as superficial-cerebellar (cortex, vermis or juxta-cortical) or deep-cerebellar (white matter, pedunculi cerebelli and gray nuclei).

**Results:**

We included 63 participants (mean age 58 years, 60% women, 42 symptomatic). In total, the 42 symptomatic patients with D-CAA had 107 symptomatic ICH (range 1–7). None of these ICH were located in the cerebellum. Six of 42 (14%, 95%CI 4–25%) symptomatic patients and none of the 21 (0%, 95%CI 0–0%) presymptomatic carriers had ≥ 1 asymptomatic cerebellar macrobleed(s). All macrobleeds were superficially located. Cerebellar microbleeds were found in 40 of 63 (64%, 95%CI 52–76) participants (median 1.0, range 0–159), 81% in symptomatic patients and 29% in presymptomatic carriers. All microbleeds were strictly or predominantly superficially (ratio superficial versus deep 15:1) located.

**Conclusions:**

Superficially located asymptomatic cerebellar macrobleeds and microbleeds are common in D-CAA. Cerebellar microbleeds are already present in the presymptomatic stage. Despite the high frequency of cerebellar micro and macrobleeds, CAA pathology did not result in symptomatic cerebellar ICH in patients with D-CAA.

## Introduction

Cerebral amyloid angiopathy (CAA) is a major cause of intracerebral hemorrhage (ICH) and cognitive decline in the elderly.^1^ Generally CAA is affecting the vessels in the cerebral hemispheres resulting in lobar ICH and strictly lobar microbleeds and affects the cerebellar vessels to a lesser extent.^2^ Recently, however, an association between symptomatic superficial cerebellar ICH and the prevalence of strictly lobar cerebral microbleeds was found.^3–5^ These findings suggest that the presence of a superficial cerebellar ICH could point to underlying CAA.^6^ This association might have clinical implications as magnetic resonance imaging (MRI)-defined CAA has a higher ICH recurrence risk which is of importance when (re)starting of oral anticoagulation is considered.^7^ Vascular amyloid deposition in the cerebellum is variable and may contribute to cerebellar hemorrhages.^8^

Definite diagnosis of sporadic CAA based on the Boston Criteria is only possible by post-mortem analysis of brain tissue.^9^ Worldwide a few hereditary forms exist that can be diagnosed by genetic testing. Dutch-type hereditary CAA (D-CAA, also known as HCHWA-D; Hereditary Cerebral Hemorrhage With Amyloidosis – Dutch type) is an autosomal dominant disease caused by a single based point mutation at codon 693 of the amyloid precursor protein gene located on chromosome 21.^10^ In D-CAA, the amyloid angiopathy is pathologically, biochemically and radiologically similar to sporadic CAA but the disease course is generally more aggressive.^11,12^ Most patients develop their first symptomatic ICH between the age of 50 to 55 years when the effect of age-related vascular risk factors is still relatively low. D-CAA can be confirmed genetically, which enables research into the early phases.

### Aims

The aim of this study was to investigate the proportion and location of symptomatic cerebellar ICH and asymptomatic cerebellar macro- and microbleeds in D-CAA.

## Methods

### Participants

We included all presymptomatic and symptomatic D-CAA mutation-carriers from our D-CAA database. This database includes all persons who have been admitted to our clinic with D-CAA-related ICH or who visited our outpatient D-CAA clinic between 1992 and April 2019. A part of the participants from our D-CAA database participated in a prospective natural history study (AURORA).

D-CAA was diagnosed by DNA analysis of the Glu693Gln mutation in the APP gene, or when patients had ≥1 symptomatic ICH with probable CAA characteristics according to the modified Boston criteria and ≥1 positive first-degree family member with D-CAA. Mutation-carriers were classified as symptomatic after occurrence of a first symptomatic ICH. We retrieved information on medical history and vascular risk factors from medical records and the D-CAA database. Patients were excluded when no MRI was available, or when the cerebellum was not fully depicted on the MRI.

This retrospective study was approved by the Medical Ethical Committee of the Leiden University Medical Hospital (LUMC), who concluded that it did not fall under the medical research on human aspects act (non-WMO).

### Radiological assessment

Two independent observers (SV and EAK) assessed the available MRI scans for CAA-related small vessel disease markers according to the STRIVE criteria.^13^ The cerebellar location was assessed with the method of Pasi et al.^3^ In case of discrepancy the results were discussed with an experienced neuroradiologist (MAAvW) to reach consensus. When multiple MRIs were available, the most recent MRI with full imaging of the cerebellum was used. MRI images were performed on 1.5 or 3 Tesla Phillips MRI scanners. Images were scored for the presence of the following CAA-related cerebellar disease markers: symptomatic cerebellar ICH, asymptomatic cerebellar macrobleeds and cerebellar microbleeds.

### Symptomatic cerebellar ICH

We analyzed the location of all symptomatic ICH and categorized them as supratentorial or infratentorial. An ICH was considered symptomatic when the participant had suffered from symptoms and/or signs which could directly be attributed to the ICH.

### Asymptomatic cerebellar macrobleeds

Asymptomatic cerebellar macrobleeds were scored using susceptibility weighted images (SWI) or T2*GE and were differentiated from symptomatic ICH by a presumed absence of neurological symptoms and/or signs. Asymptomatic cerebellar macrobleeds were differentiated from cerebellar microbleeds by either an irregular shape and/or presence of a cystic cavity.^10^

### Cerebellar microbleeds

Microbleeds were scored using SWI or T2*GE and defined according to the STRIVE consensus criteria as small areas of signal void with associated blooming, differentiated from vascular flow void and cavernomas, not visible on T1-weighted, T2-weighted or FLAIR images.^13-15^

### Location

We used the template by Pasi et al. and assessed the lesions on SWI or T2*GE sequences in combination with the T2 sequence to differentiate deep white matter from the cortical cerebellar area. Infratentorial microbleeds, asymptomatic macrobleeds and symptomatic ICH were all quantified and categorized according to location (superficial cerebellar, deep cerebellar and brainstem).^3^ Locations were considered superficial when centered in the cortex, vermis or at the junction of the cortex and cerebellar white matter. Deep cerebellar locations involved cerebellar gray nuclei, white matter and pedunculi cerebelli. The distribution pattern for cerebellar micro- and macrobleeds was considered strictly superficial when all were located superficially. In case both superficial as deep markers were present, we classified them as mixed. Within the mixed category the location was classified as predominantly superficial in case there were more superficial than deep microbleeds. If all markers were located deep and none superficial, we classified them as strictly deep.

We compared the distribution of cerebellar hemorrhages in the D-CAA participants from the prospective AURORA study with a sporadic CAA control group. This control group consisted of participants from our prospective natural history study in sporadic CAA patients (FOCAS). This study had the exact same study design and 3 T-MRI scan protocol as the AURORA study.

### Additional small vessel disease markers

In the participants of the AURORA study, we assessed markers of arteriosclerosis (enlarged perivascular spaces in basal ganglia^16^ and deep lacunes^17^) and the following additional supratentorial CAA-related MRI markers: cortical microbleeds, cortical superficial siderosis and white matter hyperintensities.

### Statistical analysis

Demographic characteristics as well as the proportion of cerebellar symptomatic ICH and macro- and microbleeds were analyzed with descriptive statistics. In patients with both cerebellar micro- and macrobleeds we compared the proportion of superficial located bleeds with deep located cerebellar bleeds. The proportion of both locations were described as the ratio of the number of superficial over deep located bleeds.

### Data availability

The dataset analyzed in this study is not publicly available because of restricted access but further information about the dataset is available from the corresponding author on reasonable request.

## Results

We retrieved 113 eligible participants from our D-CAA database. Fifty were excluded because the cerebellum was not fully scanned (n = 16) or there was no MRI performed (n = 34). We included 63 participants in the study: 42 symptomatic patients with D-CAA and 21 presymptomatic mutation-carriers. Twenty-four participated in our prospective natural history study AURORA. The mean age was 58 years (range 28–85) and 38 (60%) of them were women. Forty of the MRIs (10 on 1.5 T and 30 on 3 T) were performed in a clinical and 23 in a research setting (all 3 T). The baseline characteristics of the 63 participants are shown in Table 1.

**Table 1.** Baseline characteristics.

Characteristics of the participants (n = 63)	
*Demographics*	
Women	38 (60%)
Mean age (range, SD)	58 (28–85, 11.6)
Hypertension	29%
Hypercholesterolemia	42%
Diabetes mellitus type 2	10%
Smoking (current and/or past)	53%
Alcohol use	
Current	43%
Past	10%
Use of antithrombotic agents	0%
*Mutation status*	
Confirmed	53 (84%)
Not genetically confirmed	10 (16%)
*Symptomatic patients (≥1 ICH)*	42 (67%)
First ICH, mean age (range, SD)	56 (44–78, 8.7)
Mean number of ICH (min-max)	3 (1–7)

Hypertension was defined as ≥140/90 mmHg and/or use of antihypertensive drugs.

Hypercholesterolemia as reported in medical history and/or use of statin.

### Symptomatic ICH and asymptomatic macrobleeds

The 42 symptomatic patients with D-CAA had 107 symptomatic ICH (range 1–7). None of these ICH were located in the cerebellum.

Six (10%, 95%CI 3–17) of the 63 participants had ≥1 asymptomatic cerebellar macrobleed(s): six of 42 (14%, 95%CI 4–25%) symptomatic patients and none of the 21 (0%, 95%CI 0–0%) presymptomatic carriers. All asymptomatic macrobleeds were strictly superficially located and were <10 mm, when measured on SWI/T2* GE images (Table 2).

**Table 2.** Cerebellar hemorrhages in all, symptomatic and presymptomatic participants.

	All (n = 63)	Symptomatic (n = 42)	Presymptomatic (n = 21)
*Demographics*			
Mean age (range, SD)	58 (28–85, 11.6)	62 (48–85, 8.9)	49 (28–69, 11.3)
Women (%)	38 (60%)	24 (57%)	14 (67%)
*Cerebellar ICH*	*0/107*	*0/107*	–
*Asymptomatic cerebellar macrobleeds*	*6 (10%)*	*6 (14%)*	–
Median number of macrobleeds (range)	0 (0–14)	0 (0–14)	
*Location superficial*	*6 (100%)*	*6 (100%)*	
Median number of superficial (range)	0 (0–14)	0 (0–14)	–
*Location deep*	*0 (0%)*	*0 (0%)*	
Median number of deep (range)	0 (0–0)	0 (0–0)	–
*Distribution pattern*			
Strictly superficial, n (%)	6 (100%)	6 (100%)	–
Strictly deep, n (%)	0 (0%)	0 (0%)	–
Mixed, n (%)	0 (0%)	0 (0%)	–
*Cerebellar microbleeds*	*40 (64%)*	*34 (81%)*	*6 (29%)*
Median number of microbleeds (range)	1.0 (0–159)	2.0 (0–159)	0.0 (0–1)
*Location superficial*	*40 (63%)*	*34 (81%)*	*6 (29%)*
Median number of superficial (range)	1 (0–154)	2 (0–154)	0 (0–14)
*Location deep*	*11 (17%)*	*10 (24%)*	*1 (5%)*
Median number of deep (range)	0 (0–5)	0 (0–5)	0 (0–1)
*Distribution pattern*			
Strictly superficial, n (%)	29 (73%)	24 (71%)	5 (83%)
Strictly deep, n (%)	0 (0%)	0 (0%)	0 (0%)
Mixed, n (%)	11 (27%)	10 (29%)	1 (17%)
Mean ratio (superficial:deep)^a^	14:1	15:1	5:1

a Mean ratio is based on participants with microbleeds.

### Cerebellar microbleeds

**Figure 1.**
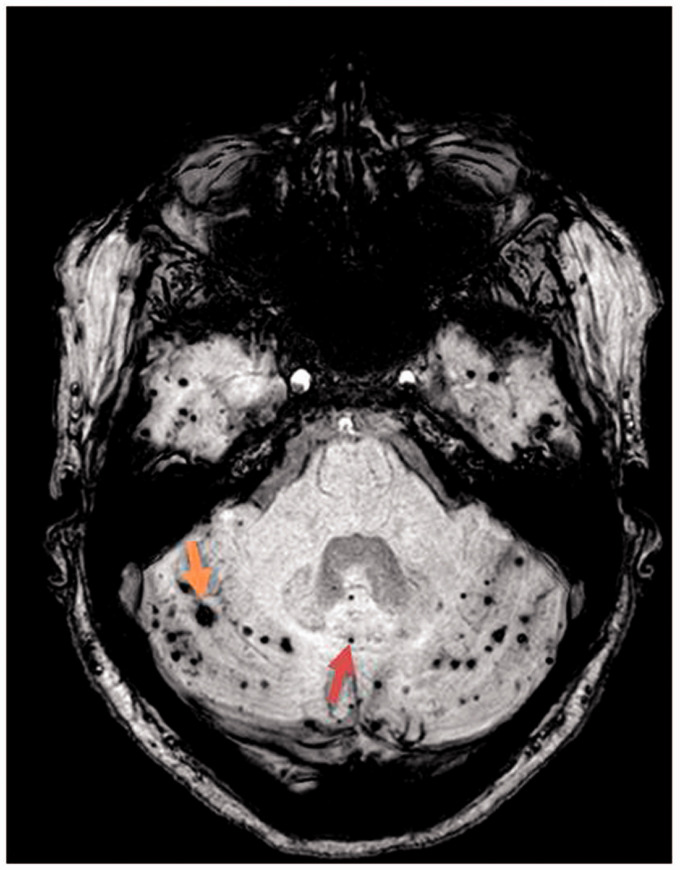
Example of a symptomatic case (n=3 previous ICH) with multiple cerebellar hemorrhages.

Forty (64%, 95%CI 52–76) of the 63 participants had one or more cerebellar microbleeds: 34 (81%) of the 42 symptomatic patients and 6 (29%) of the 21 presymptomatic mutation-carriers (Table 2) (Figure 1). Cerebellar microbleeds were only found in the presymptomatic carriers over the age of 50 (6/10 of the carriers ≥50 years, n = 3 in 50–55 group and n = 3 in 65–70 group) and in none of the 11 presymptomatic carriers younger than 50 years. In 29 (73%) of the participants with cerebellar microbleeds, the distribution was strictly superficial in location (71% in symptomatic patients and 83% in presymptomatic mutation-carriers). In 10 symptomatic patients the microbleeds were mixed but predominantly superficial with a mean ratio of superficial-deep of 15:1. Three (27%) participants with deep cerebellar microbleeds had hypertension, versus 11 (21%) participants without deep cerebellar microbleeds. None of the participants had a strictly deep distribution of cerebellar microbleeds. The distribution of cerebellar microbleeds was similar in the sporadic CAA control group (Table 3). All of the patients with D-CAA with cerebellar microbleeds had supratentorial microbleeds, versus 36% of patients without cerebellar microbleeds (Table 4).

**Table 3.** Cerebellar MRI markers in a subset of patients with D-CAA and patients with sporadic CAA.

	Patients with D-CAA (n = 24)	Patients with sporadic CAA (n = 31)
*Demographics*		
Mean age (range, SD)	53 (28–75, 12.2)	72 (57–86, 6.6)
Women (%)	13 (54%)	16 (52%)
*Infratentorial markers*		
*Cerebellar ICH*, n (%)	*0 (0%)*	*0 (0%)*
*Asymptomatic* c*erebellar macrobleeds*, n (%)	*1 (4%)*	*6 (19%)*
Location superficial, n (%)	1 (100%)	6 (100%)
Location deep, n (%)	0 (0%)	0 (0%)
*Cerebellar microbleeds*, n (%)	*11 (46%)*	*11 (33%)*
Location superficial, n (%)	11 (100%)	11 (100%)
Location deep, n (%)	3 (0.3%)	1 (0.1%)

**Table 4.** Supratentorial MRI markers in a subset of patients with D-CAA.

	Patients without cerebellar microbleeds (n = 11)	Patients with cerebellar microbleeds (n = 10)
**Supratentorial markers**		
*Cortical microbleeds*, n (%)	*4 (36%)*	*10 (100%)*
<10	1 (9%)	0 (0%)
10–50	1 (9%)	2 (20%)
50–100	2 (18%)	1 (10%)
>100	0 (0%)	7 (70%)
*Cortical superficial siderosis*, n (%)	*2 (18%)*	*3 (30%)*
*Periventricular WMH Fazekas scale*, n (%)		
0	4 (36%)	0 (0%)
1	3 (27%)	1 (10%)
2	1 (9%)	3 (30%)
3	3 (27%)	6 (60%)
*Deep WMH Fazekas scale*, n (%)		
0	5 (46%)	0 (0%)
1	3 (27%)	1 (10%)
2	2 (18%)	5 (50%)
3	1 (9%)	4 (40%)

WMH = White Matter Hyperintensities.

The interobserver variation (Kappa statistic) for cerebellar microbleeds was almost perfect (0.96).

Patients with D-CAA had only minimal signs of deep perforating arteriopathy. We found no deep lacunes and 92% of the patients with D-CAA had score 1 (1–10) of enlarged perivascular spaces in the basal ganglia.

None of the participants with D-CAA who had cerebellar micro and/or macrobleeds had symptoms that could be related to the cerebellum. Results remained the same after exclusion of the n = 10 probable D-CAA carriers.

## Discussion

Our study shows that superficial cerebellar macrobleeds and microbleeds are common in patients with D-CAA and cerebellar microbleeds are also present in the presymptomatic stage. Surprisingly, in none of the participants (remnants of) symptomatic cerebellar ICH were found despite the large number of symptomatic ICH.

D-CAA is pathologically, biochemically and radiologically identical to sporadic CAA and considered to be a model for CAA. The lack of symptomatic cerebellar ICH in D-CAA could indicate that cerebellar ICH in sporadic CAA is more likely based on a mixed type small vessel disease or is triggered by other ageing-related factors. Pathological studies in sporadic CAA are needed to further investigate this hypothesis.

Eight out of every 10 symptomatic and 2 out of every 10 presymptomatic mutation-carriers had cerebellar microbleeds. The microbleeds were predominantly superficially located, which confirms recent findings in sporadic CAA that superficial cerebellar microbleeds are associated with CAA.^3,4^ In a recent study, the prevalence of strictly superficial cerebellar microbleeds was 47% in patients with deep/mixed ICH and 35% in patients with ICH caused by probable CAA.^4^ This proportion of 35% is lower than that we found in our genetically affected patients who, in general, have a more aggressive course of CAA compared with the sporadic patients.

In the presymptomatic mutation-carriers, cerebellar microbleeds were only present in the relatively older (≥50 years, mean age 60 years) participants. Previously we found that white matter hyperintensities and microinfarcts are the first signs of CAA in young mutation carriers.^18^ Superficial cerebellar microbleeds are, therefore, probably more a mid-phase characteristic of D-CAA.

The proportion of strictly superficial microbleeds was 95% in presymptomatic mutation-carriers and 57% in symptomatic patients. This discrepancy could be explained by the higher age of the symptomatic patients leading to a higher prevalence of hypertension or diabetes and thus a higher chance of also accumulating deeply located microbleeds. A previous D-CAA study found that mutation-carriers with hypertension had more microbleeds in the cerebellum than mutation carriers without hypertension.^19^ However, the mean ratio of 15:1 superficially-deeply located microbleeds (lowest ratio 1:1) in our study suggests that the contribution of deeply located microbleeds is limited.

We only detected asymptomatic cerebellar macrobleeds in symptomatic patients and not in presymptomatic carriers. This suggests that the formation of asymptomatic cerebellar macrobleeds occurs in the same period or after the formation of cerebral microbleeds. The pathophysiology and the prognostic meaning of macrobleeds is largely unclear. The macrobleeds in our study were classified according to the Strive criteria. Despite the classification “macro” these lesions were always smaller than 10 mm, when measured on SWI/T2* GE images. However, all were either irregular shaped and/or had a cystic cavity and, therefore, clearly differed from cerebral microbleeds. Our study has the following limitations. First, due to a retrospective design, scans were performed with different protocol settings and field strengths. Although this might have resulted in an underrepresentation of microbleeds in participants scanned on 1.5 T MRI, it is unlikely that the differences in field strength affected the proportion superficial versus deep microbleeds, the number of macrobleeds or the assessment of ICH location. Second, we focused on D-CAA and did not include a sporadic CAA control group. Therefore, we cannot evaluate whether the pattern of micro or macrobleeds in our study are general for CAA or specific for this particular hereditary form. Third, selection bias is a risk in studies on cerebellar ICH as large hemorrhages have a poor prognosis with high risk of herniation due to marginal space in the posterior fossa.^20^ In our hospital, in the acute setting, mostly computer tomography instead of MRI is performed. We, therefore, post-hoc, assessed all available computer tomography scans of the 50 patients with D-CAA we excluded but none of them had cerebellar ICH (data not shown) making selection bias unlikely. Lastly, 16% of our symptomatic patients were not genetically tested. However, all of them had at least one lobar ICH in combination with CAA characteristics on MRI and a positive first-degree family history of D-CAA.

Strong points are our unique hereditary CAA population including presymptomatic carriers, which makes it possible to investigate the early stages of disease. In addition, because of the relatively young age of presentation, the effect of age-related vascular risk factors is limited making D-CAA a relatively pure form of CAA.

Future research is necessary to investigate whether strictly cerebellar macro and micro hemorrhages have a prognostic meaning and can contribute to the modified Boston criteria for diagnosing CAA.
